# Response of the Salmon Heart Transcriptome to Pancreas Disease: Differences Between High- and Low-Ranking Families for Resistance

**DOI:** 10.1038/s41598-020-57786-1

**Published:** 2020-01-21

**Authors:** N. A. Robinson, A. Krasnov, E. Burgerhout, H. Johnsen, H. K. Moghadam, B. Hillestad, M. L. Aslam, M. Baranski, S. A. Boison

**Affiliations:** 1 Breeding and Genetics, Nofima, Ås 1430 Norway; 2 Sustainable Aquaculture Laboratory- Temperate and Tropical (SALTT), School of BioSciences, The University of Melbourne, Parkville, 3010 Australia; 3 Benchmark Genetics, Bergen, 5035 Norway; 4 Mowi Genetics AS, Bergen, 5835 Norway

**Keywords:** Agricultural genetics, Transcriptomics

## Abstract

Pancreas disease caused by salmonid alphaviruses leads to severe losses in Atlantic salmon aquaculture. The aim of our study was to gain a better understanding of the biological differences between salmon with high and low genomic breeding values (H-gEBV and L-gEBV respectively) for pancreas disease resistance. Fish from H- and L-gEBV families were challenged by intraperitoneal injection of salmonid alphavirus or co-habitation with infected fish. Mortality was higher with co-habitation than injection, and for L- than H-gEBV. Heart for RNA-seq and histopathology was collected before challenge and at four- and ten-weeks post-challenge. Heart damage was less severe in injection-challenged H- than L-gEBV fish at week 4. Viral load was lower in H- than L-gEBV salmon after co-habitant challenge. Gene expression differences between H- and L-gEBV manifested before challenge, peaked at week 4, and moderated by week 10. At week 4, H-gEBV salmon showed lower expression of innate antiviral defence genes, stimulation of B- and T-cell immune function, and weaker stress responses. Retarded resolution of the disease explains the higher expression of immune genes in L-gEBV at week 10. Results suggest earlier mobilization of acquired immunity better protects H-gEBV salmon by accelerating clearance of the virus and resolution of the disease.

## Introduction

Outbreaks of pancreas disease (PD) caused by salmonid alphavirus (SAV) affect farmed and wild populations of Atlantic salmon (*Salmo salar*) and other salmonids in many places around the world. Outbreaks of PD lasting 3–4 months regularly occur in Norway and are expensive, with direct costs of a single PD outbreak at a site stocked with 1 million salmon smolts estimated at Euro 7 million^1–8^. Monitoring and control is difficult as there are often no obvious external signs of infection (sub-clinical infections^9,10^), and because an estimated one-third of SAV infected populations in Norway do not develop clinical PD^11^. DNA^12^ and inactivated whole virus^13^ vaccines have been found to be effective but do not provide complete protection.

The virus causes severe loss of pancreatic tissue by necrosis, heart inflammation and necrosis of cardiomyocytes in post-smolt salmon (the heart recovering quickly after week 4 of infection, the pancreas taking longer to recover and still not fully recovered by week 12^14^) and this affects the animal’s physiology resulting in loss of appetite, slow swimming near the surface at the corners of the sea cage or resting on the bottom^8,15,16^. Suppression of the immune transcriptome during smoltification^17^ can be partly responsible for higher susceptibility to PD during the first several months in the sea, when mortality can reach up to 80%^18–20^. The SAV virus normally persists up until slaughter even if fish were infected during early seawater phases^21^. There are several subtypes of the virus, with subtype-3 (SAV3) found in mid and southern Norway^3,22^.

Heritability of resistance to PD is moderate to high (~0.4 in post-smolts, ~0.5 in fry)^23,24^, and quantitative trait loci (QTL) affecting resistance have been detected^25^, so that resistance to the disease could be achieved with selective breeding. At present, little is known about the biological basis for genetic resistance to the disease. Here we aimed to detect genes and pathways associated with PD resistance. We compared the transcriptome of the heart of Atlantic salmon with high and low resistance to PD, based on estimated genomic breeding values (gEBV), before and after experimental challenge with SAV3. Two challenge models (co-habitation and intraperitoneal injection of SAV3) were compared and assessed.

## Method

### SAV3 challenge test

Genomic family breeding values (gEBV’s) for pancreas disease resistance were estimated by SalmoBreed AS (Bergen, Norway) from parental PD survival data generated for the company’s in-house selective breeding program. The survival data was collected from smolts that were challenged with intraperitoneal injection of SAV3. Fish tagged with passive integrated transponders (PIT-tags), from families that had high-gEBV’s or low-gEBVs (H-gEBV and L-gEBV respectively) for PD survival (point deviation from mean gEBV of +27.3% and −14.3% for the high- and low- families, respectively), were selected and contributed by SalmoBreed AS in August 2016.

**Figure 1:**
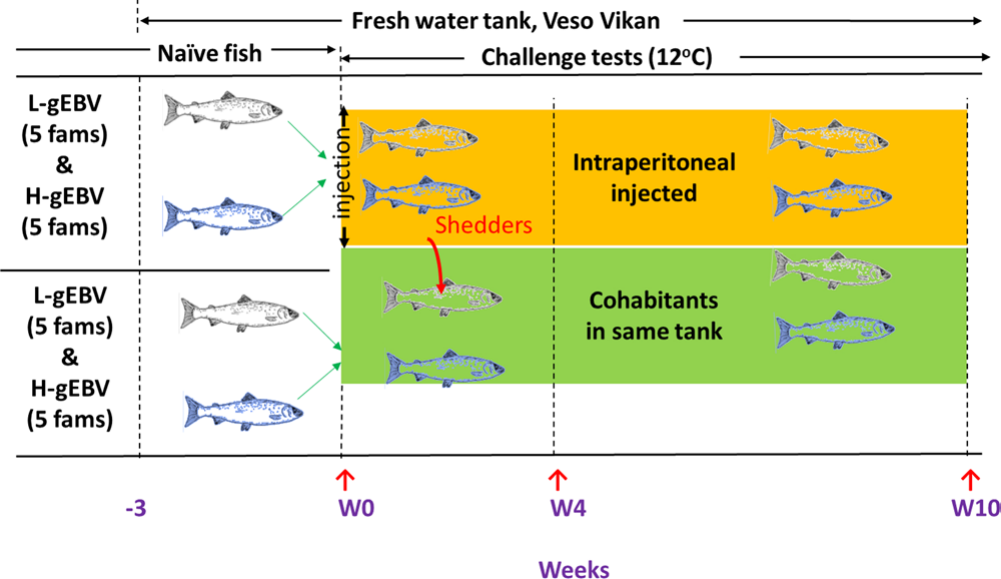
Procedure followed for the 10-week challenge test trial. Red arrows indicate when heart was sampled from some of the fish for RNA extraction and histology: immediately before challenge (W0); 4 weeks post-challenge (W4), and; 10 weeks post-challenge (W10). Intraperitoneal injected fish (IP challenge model) from 5 families of both low and high genomic estimated breeding value groups for PD resistance (L-gEBV and H-gEBV, respectively) were used as shedders for the cohabitant challenge model (CH). Shedding is assumed to have occurred after W0 over the duration of the experiment.

The study started with 1345 unvaccinated parr stage Atlantic salmon with an average weight of 37 g, consisting of more than 110 fish from each of five H-gEBV and five L-gEBV families (Fig. 1). The fish sent for challenge testing were issued with a standard health certificate before transport (specific tests for PRV-1 were not performed). The fish were acclimatised in one fresh water tank at 12 °C for 3 weeks, and starved for 24 hours, before some were challenged with SAV3 (as described below). Fish were stocked at a density of 40 kg/m^3^, fed by automatic feeders and maintained on a 24-hour light regime. The tank was cleaned every day and water was maintained at 12 °C ± 1 °C with a flow of 0.8 L per kg of fish per minute. Dead fish were collected, and mortalities recorded daily throughout the experiment.

Heart ventricle was chosen as a target tissue for the study as it is severely damaged by the disease during infectious stages (more severely than pancreas tissue), but later recovers^8,15,16^. A total of 115 fish were euthanised (standard operating procedures at VESO) and their hearts were sampled one day before the challenge (designated week 0, W0, Table 1). The 1200 remaining fish were anaesthetised, PIT tags scanned and half (600, consisting of an equal number of representatives from each family) were challenged by intraperitoneal injection (IP group) with 0.1 ml of SAV3 suspension (isolate VESO Vikan Jnr. 2333 at 2.5 × 10^7^ virus/ml) and transferred to the challenge tank. The choice of dose of the virus for the challenge was based on several previous PD challenge trials performed at VESO Vikan accounting for the size of the fish, total biomass and rate of water exchange.

**Table 1:** Numbers of animals sampled for RNA extraction. Numbers of samples used for mRNA-seq is shown in parentheses.

gEBV	Challenge model	Naïve (W0)	Challenge W4	Challenge W10	Total
High	IP	55 (15)	140 (5)	131 (5)	
	CH		141 (5)	122 (5)	
Low	IP	60 (15)	116 (5)	131 (5)	
	CH		85 (5)	107 (5)	
**Total**		**115 (30)**	**482 (20)**	**491 (20)**	**1088 (70)**

At the same time, the other 600 fish (co-habitants, ie. CH challenge model) were anesthetised (standard operating procedures at VESO), PIT tags scanned and transferred to the same challenge tank as the IP group fish. This was done to check the PIT tag numbers of the remaining fish, and so that both IP and CH challenge fish were treated in the same way. The IP group fish served as shedder fish for the CH challenge model (IP infected fish shed virus into the water during viraemia 4–13 days after infection^26^). A total of 482 fish were euthanised and their hearts were sampled 4 weeks (W4) post-challenge (Table 1). Due to tank size and biomass, the remaining fish were moved into a new tank after sampling at W4. At week 10 (W10), surviving fish were euthanised and their hearts were sampled (Table 1). Mortalities were registered daily (except during W4 sampling days) along with PIT tag numbers and date. Survival curves were generated by first removing from the data set all fish that had died of causes other than PD (eg. fish that jumped out of the tank). The animals sampled at the termination of the experiment (W10) were included in all percent survival calculations. Up until day 28, the surviving animals also included those that were subsequently sampled on day 28 (W4). After day 28, percent change in survival was estimated (removing W4 sampled animals from the pool for consideration). The change in percent survival at each day after day 28 was deducted from the level of percent survival reached at day 28.

All challenge testing procedures were performed by VESO Vikan in a secure containment facility in Namsos Norway using standard operating procedures as approved by the Norwegian Animal Research Authority, National Assignments Department (approval no. 8841). Both challenges were conducted in accordance with the laws and regulations controlling experiments and procedures for live animals in Norway (the Animal Welfare Act of December 20th 1974, No 73, chapter VI sections 20–22 and the Regulation on Animal Experimentation of January 15th 1996). The challenge testing procedure employed standard protocols that had been previously developed by VESO for the salmon selective breeding companies of Norway. This same protocol is routinely used by VESO to provide data for evaluating breeding values for disease resistance by breeding companies and research institutes of Norway.

### Sampling for RNA extraction

Immediately after euthanasia, approximately 30–50 mg of atrium and bulbous heart tissue was sampled for RNA extraction (numbers of fish are shown in Table 1). Heart tissue for RNA extraction was cut into 2–3 pieces and stored in RNALater (per manufacturer’s specifications, Ambion). All 1088 samples were used in qPCR analyses. At W0, hearts from three randomly selected naïve fish derived from each of the five H-gEBV and each of the five L-gEBV families for PD resistance were sampled for mRNA-seq from the pool of samples. One randomly selected fish from each of the five H-gEBV and five L-gEBV families that were subjected to each challenge model at W4 and W10 post-challenge were also used for mRNA-seq. This resulted in a total of 15 H-gEBV and 15 L-gEBV naïve fish at W0 and 5 fish per EBV ranking and challenge model at W4- and W10 post-infection.

### Sampling for histology

Half of the ventricle was sampled in formalin from six naïve fish per family in W0, and 12 fish per family at W4 and W10 post-infection (i.e. six fish from the IP and six from the CH challenge model). These fish were a subset of the same fish that were sampled for RNA extraction (Table 1). Heart lesions were scored based on the severity of focal acute myocardial degeneration and inflammation (as detailed in Table 2, a semiquantitative scoring system adapted from^27^) by an experienced histopathologist at the Fish Vet Group in Oslo Norway. Data was analysed using an ordinal logistic regression (polr module in r^28^).

**Table 2:** Scores of heart lesions.

Score	Damage
0	Normal appearance (no degeneration or inflammation)
1	Less than 7 fibres were affected
2	Less than 15% of the ventricle affected
3	More than 15% and less than 50% of the ventricle was affected
4	More than 50% of the ventricle was affected

### Real-time qPCR

Total RNA was extracted using a MagNA Pure 96 Cellular RNA Large volume kit (Roche Life Science) and the MagNA Pure 96 Instrument according to the manufacturer’s protocol. Quality and quantity of the RNA was assessed using a 2000c Nanodrop spectrophotometer (Thermo Scientific). RNA with a 260/280-ratio >1.8 was used to synthesise cDNA using the Quantabio qScript cDNA SuperMix (Quantabio) using 10 µL RNA template and 10 µL SuperMix. Genes that showed strong responses to PD in previous studies^29,30^ were selected, taking into account their functional roles (Table 3): innate antiviral immunity (*sacsin*), humoral and cellular inflammatory responses (*serum amyloid A* and *matrix metalloproteinase 13*) and tissue protection and repair (*neuropeptide Y*). *Beta actin* was used as a reference gene. To determine the PCR efficiency (%), a two-fold standard dilution of heart cDNA was tested for each primer set. The RT-qPCR was run in duplicates on a QuantStudio 5 system (Applied Biosystems, Thermo Fisher Scientific). Reactions had a total volume of 20 µL consisting of 10 µL Power SYBR Green PCR Master Mix (Applied Biosystems), 0.6 µL 10 µM forward and reverse primers, 8 µL 1:40 diluted cDNA. The cycling profile was 2 min at 50 °C and 10 min at 95 °C, followed by 40 cycles at 95 °C for 15 s and 60 °C for 60 s. A no-template (water) control was included to rule out non-specific contamination and a melting curve analysis was performed to verify the measurement of a single specific product. QuantStudio Design & Analysis Software (Applied Biosystems, Thermo Fisher Scientific) was used for data collection and analysis. Relative gene expression was calculated using cycle threshold (Ct) values as deltaCt (Ct target gene – Ct reference gene) and results for SAV are presented as -Ct normalized by RNA concentration. Data were analysed with ANOVA followed with Newman – Keuls test.

**Table 3:** Overview of RT-qPCR primer sequences, PCR efficiency, accession number (NCBI Genbank) and references to primers designed (when from the literature) for the selected genes involved in PD.

Gene	Sequence 5′ → 3′	PCR eff.	Accession nr.	Reference
sacsin	Fwd: CACAGAGTGGGCTCTGTTCA	103.6%	EG906096	—
	Rev: CTCAGCTAGGCTGGAGATGG			
matrix metalloproteinase 13	Fwd: AGTGTCCAGCACAAATGACCT	97.1%	XM_014163130.1	^30^
	Rev: CTCAACTGCTGATCCACTGGT			
neuropeptide Y1	Fwd: GCTACCCGGTCAAACCTGAA	102.4%	XM_014178359.1	^30^
	Rev: GGACTGTGGGAGCGTATCTG			
serum amyloid A5 protein	Fwd: GGTGCTAAAGACATGTGGCG	94.0%	NM_001146565.1	^30^
	Rev: CCACTGGAACCCTGAACCAT			
salmonid alphavirus	Fwd: CCGGCCCTGAACCAGTT	103.1%	AY604235	^60^
	Rev: GTAGCCAAGTGGGAGAAAGCT			
β-actin (reference)	Fwd: CAGCCCTCCTTCCTCGGTAT	NA	BT059604	^61^
	Rev: CGTCACACTTCATGATGGAGTTG			

### mRNA-seq

mRNA-seq was performed on an Illumina platform to detect differential gene expression associated with breeding value at time points before and after the experimental challenge tests. Multiplexed libraries were prepared from 300–1000 ng input total RNA using the TruSeq Stranded mRNA reagents (Illumina, San Diego, CA). Six lanes of a HiSeq. 3000 (Illumina) was used to sequence the 70 multiplexed libraries for 150 bp paired-end reads according to manufacturer’s instructions. Illumina’s RTA software version 2.7.7 was used for image analysis and base calling, with de-multiplexing and fastq generation using Bcl2fastq version v2.18.0.12. Reads were filtered to remove those with low base call quality using Illumina’s default chastity criteria. All raw sequences have been deposited in the NCBI Short Read Archive under BioProject ID PRJNA543940.

### Differential gene expression analysis

Trimmomatic^31^ was used to trim sequencing reads of adapters and low-quality base regions. Leading and trailing sequences with quality scores of less than 15 were removed and sequences were trimmed when the average quality per base within a four base sliding window over the sequence length was less than 15 or three respectively. Finally, trimmed and filtered reads with lengths less than 30 bases were removed. Short reads were mapped to Atlantic salmon genome assembly GCA000233375.4 ICSASG_v2^32^ using TopHat version 2.0.8b^33^ with Bowtie1^34^. Default parameters for TopHat were used except for the *b2* option *sensitive*^34^. The *scripts*.*count* python program in HTSeq (version 0.8.0^35^) was used to count the reads in the bam file mapping to each feature (ordering according to name, skipping reads with alignment quality less than 10, using *exon* as the feature type, *gene_id* as feature ID and *union* mode to handle reads overlapping more than one feature). Differential gene expression was tested using a model for analysis of read coverage data based on the negative binomial distribution (DESeq. 2 package in R)^36^. Genes with expression differences between L-gEBV and H-gEBV were selected by criteria: >1.75-fold and p < 0.05. A gene enrichment analysis was performed by comparing the proportion of differentially expressed genes assigned to each GO annotation for each contrast to the overall assignment of the full set of genes expressed in all states (Fisher’s exact test, topGO package in R)^37^.

## Results and Discussion

This study provides useful new insights into the biology of PD resistance in Atlantic salmon. By comparing H-gEBV to L-gEBV fish for PD resistance we identified genes and pathways that come into play after infection, and identified differences that could contribute to the inherent variation in resistance to PD previously observed^23,24^. The findings will be used to help us develop genomic tools that can be used for breeding to increase resistance to PD in Atlantic salmon.

### Contrasts between challenge test models

**Figure 2:**
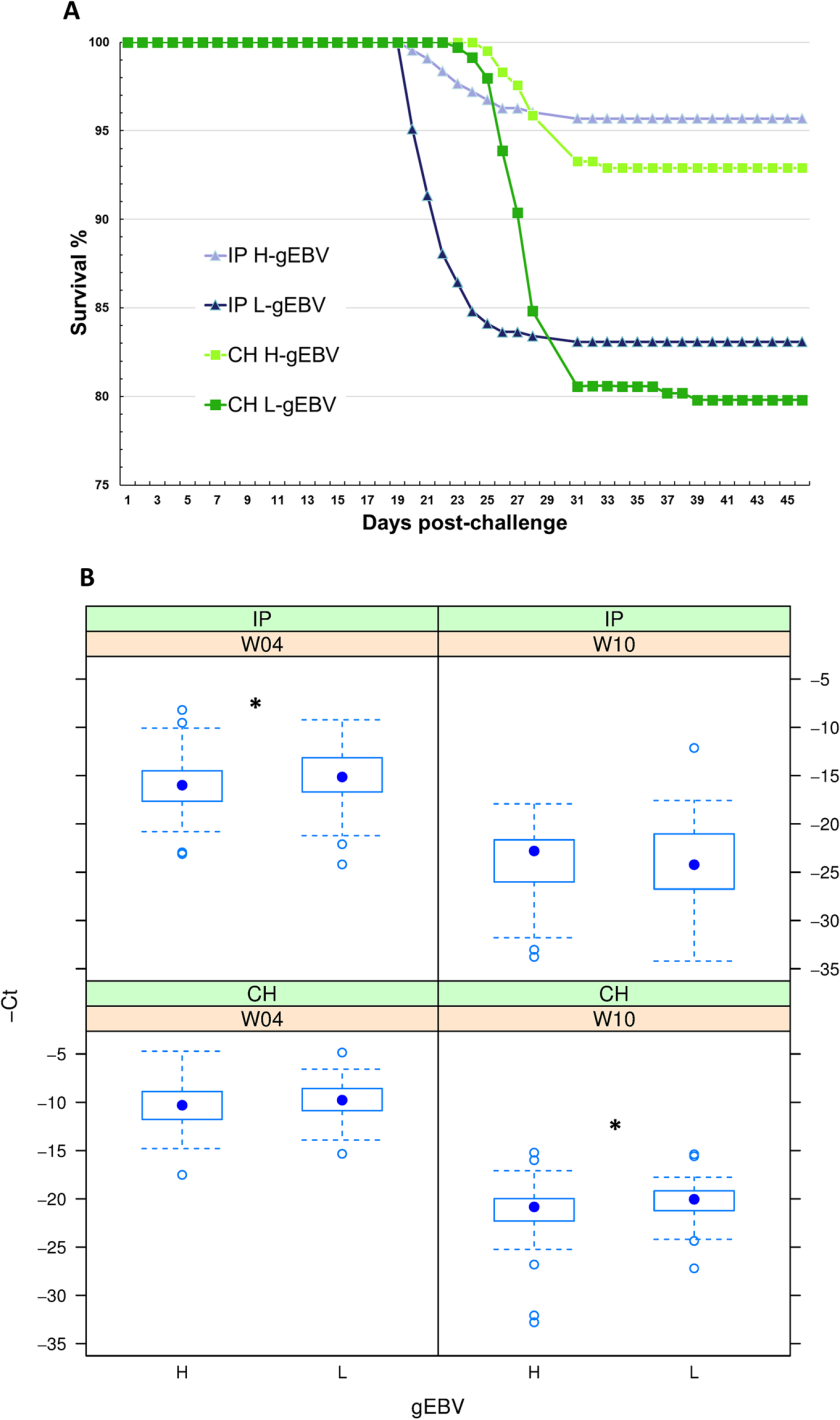
SAV3 challenge tests. (**A**) Survival over days post-challenge for the intraperitoneal (IP) and cohabitant (CH) challenge test models for high and low genomic breeding value fish (H-gEBV and L-gEBV respectively). (**B**) Boxplot of negative cycle threshold values (-Ct) from RT qPCR of SAV3 (*P < 0.05). The groups are denoted by gEBV (H for high, L for low) and time-points (at W4 post-challenge and W10 post-challenge) for challenge models (IP for intraperitoneal injection and CH for co-habitant). The point sampled for W4 corresponds to day 28 of the survival curve.

The study employed IP and CH challenge models, which are widely used, each having their own relative advantages and drawbacks. Injection ensured that a relatively equal dose of the virus was delivered to each fish and that the time of infection was precisely known. In contrast, CH employed a natural route of infection mimicking the infection pathway observed naturally in aquaculture. From other studies we expected that IP infected fish would shed virus into the water during viraemia 4–13 days after infection^26^. Due to these differences the pattern of mortalities and physiological reaction of the fish differed under the two challenge models. Mortality was observed during short periods in both challenges: 21–26 days post challenge in IP and 25–29 days post challenge in CH challenge, the mortality period caused by CH lagging behind that caused by IP by 4 days (Fig. 2A). While mortality was higher for CH challenged fish, differences in survival between H-gEBV and L-gEBV were similar (13%) in both challenge models (survival 96% versus 83% in IP and 93% versus 80% in CH respectively). As there were no signs of other infections among the fish used in our study we have assumed that the influence of side-infections on the results could be considered as negligible.

**Figure 3:**
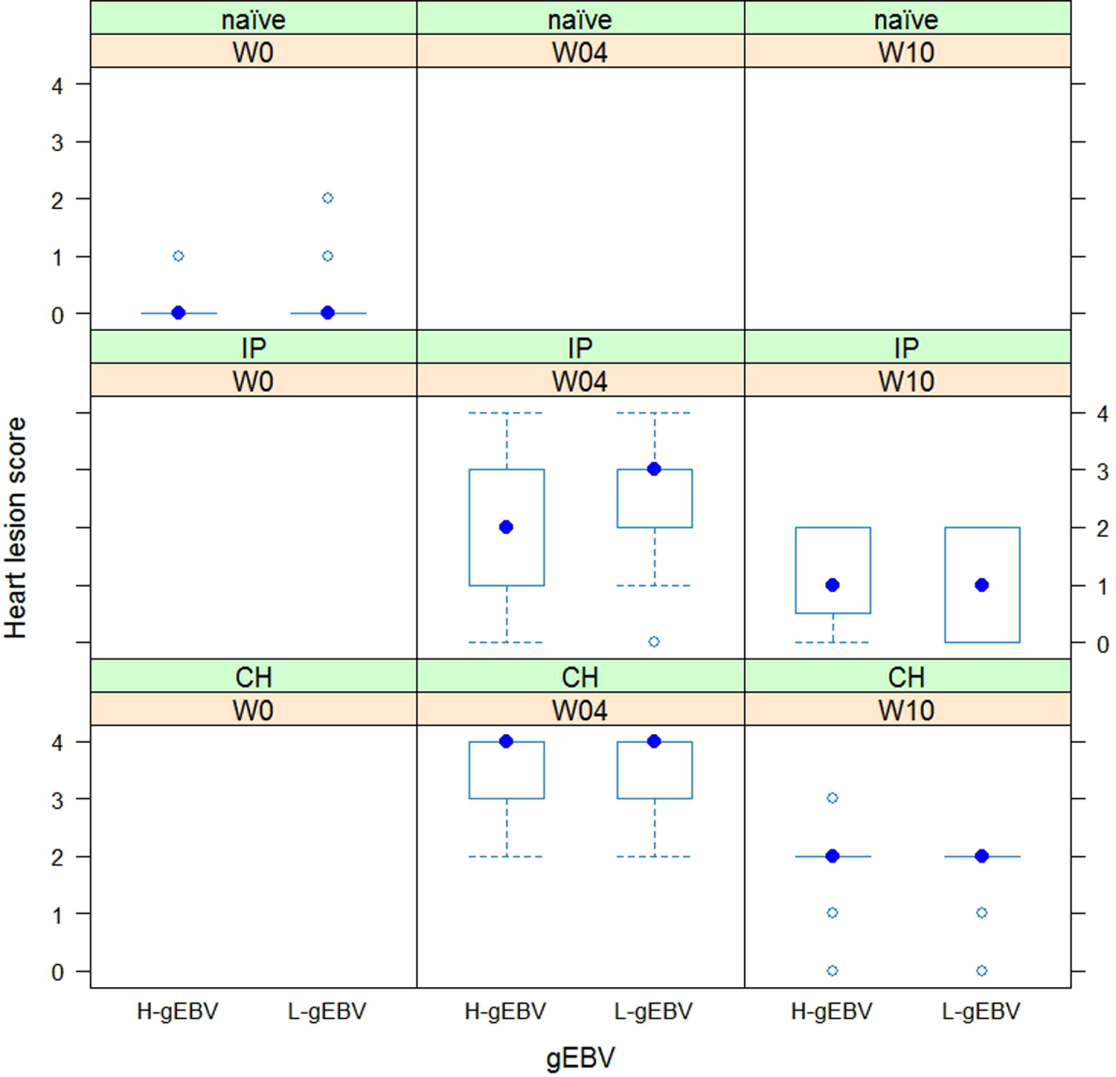
Histopathology of the heart for naïve fish and over the time course of infection for intraperitoneal (IP) and cohabitant (CH) challenge models. Heart lesion scores were based on the severity of focal acute myocardial degeneration and inflammation where 0 was normal appearance (no degeneration of inflammation), 1 when less than 7 fibres were affected, 2 when less than 15% of the ventricle affected, 3 when more than 15% and less than 50% of the ventricle was affected and 4 when more than 50% of the ventricle was affected. Results for fish with high or low genomic breeding values (H-gEBV or L-gEBV respectively) are shown in panels to the left and right respectively (showing significantly more severe lesions for L-gEBV fish compared to H-gEBV fish at W4 post-IP challenge, Ordered Logistic Regression with P < 0.05).

SAV3 levels (and mortality levels) were higher in CH- than the IP-challenged salmon at W4 (by around 5.5 Ct units). SAV3 levels were reduced at W10 indicating clearance of the pathogen (difference between the time-points was 9.56 Ct units, Fig. 2B). This was most likely due to the different course of the infection and disease – samples were collected from both the CH and IP challenged fish in weeks 4 and 10 (W4 and W10), and these time points corresponded to different phases of PD infection under the two different challenge test models. The virus load for the IP challenged fish at W4 was intermediate between the levels observed at W4 and W10 for CH. In two of four comparisons between the H- and L-gEBV salmon groups, the SAV3 levels were lower in H-gEBV, the difference being small (less than one Ct unit) but significant (*P* < 0.05). In concordance with the SAV virus *C*_*t*_, average heart lesion scores started at zero in naïve fish (indicating normal appearance), increased to average lesion scores of 2–4 at W4 post-infection (indicating moderate to severe damage) and eased to average lesion scores of 1–2 at W10 post-infection (indicating recovery, Fig. 3). Only a few fish had some background inflammation (Fig. 3, some with less than 7 fibres affected and some fish with less than 15% of the ventricle affected). Damage at W4 and W10 was highest for the CH challenge model (average severity of damage at W4 post-cohabitant challenge was extreme, with more than 50% of the ventricle affected). For the IP challenge, the severity of damage was significantly higher for L-gEBV fish than H-gEBV fish at W4 post-challenge (Ordered Logistic Regression, *P* < 0.05).

**Figure 4:**
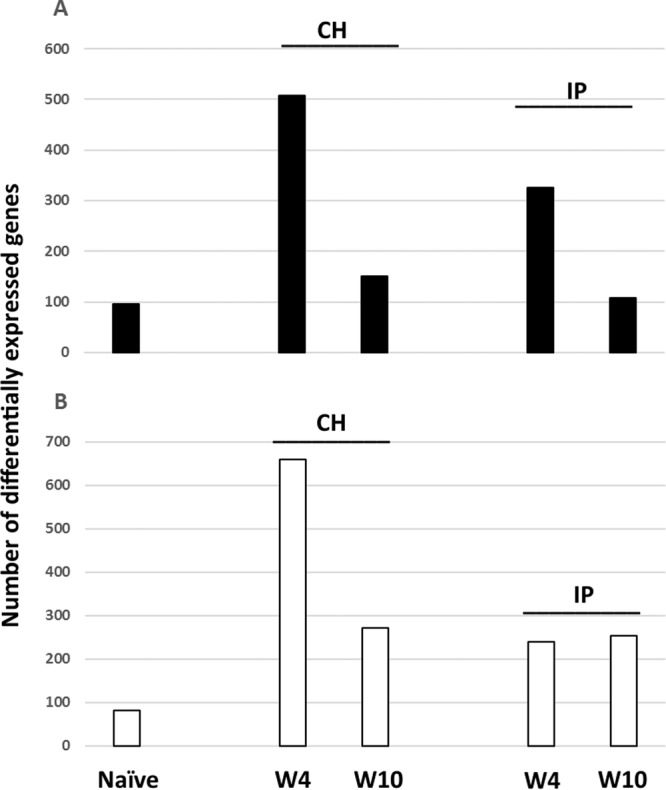
The number of differentially expressed genes in high compared to low genomic breeding value salmon (H-gEBV and L-gEBV respectively) for time points W0 (naïve), W4 and W10 post-infection under CH and IP challenge models. (**A**) Black shaded bars represent genes with higher expression in H-gEBV fish. (**B**) White shaded bars represent genes with lower expression in H-gEBV fish. Genes were selected by criteria: >1.75-fold difference in expression, p < 0.05.

The numbers of genes with expression differences between H-gEBV and L-gEBV increased after challenge at W4, and decreased at W10, but remained greater than in naïve fish (Fig. 4). The contrast between the number of genes differentially expressed between the H- and L-gEBV salmon groups was greater in the CH trial. Many immune genes changed profiles from higher expression in H-gEBV than L-gEBV fish at W4, to lower expression in H-gEBV than L-gEBV fish at W10 (Supplementary Table 1).

In this study the CH model was generally more informative for comparing the transcriptional differences between the salmon families with different breeding values (e.g. higher number of differentially expressed genes at W4 post-infection, Fig. 4). This could be due to differences in the timing of sampling relative to the timing of infection and immune response under the different challenge models (W4 occurring during the period of highest mortality and high SAV3 load for the CH challenge, Fig. 2). Differences between H-gEBV and L-gEBV in mortality and viral loads were similar under both challenge models. Because the gEBVs were calculated based on an IP challenge test model, selection would most likely favour fish with a better ability to defend against viruses that have already passed external barriers to infection (i.e. mucous or mucosal layers of the skin, gut, gills, eyes etc.). The use of CH models in breeding programs may be more effective for improving the overall resistance of the fish, as they would combine selection for more effective barriers that protect against infection by the virus with selection for the ability to respond to and clear infection once the barrier is breached^38^.

### Differential gene expression in naïve fish

**Figure 5:**
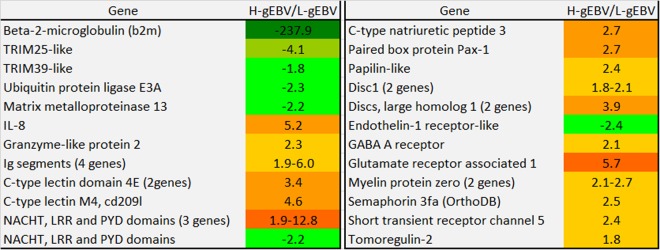
Highlighted genes of interest that were differentially expressed in naïve fish. In this and subsequent figures, data are for high to low genomic breeding value ratios (H-gEBV/L-gEBV, fold differences, colour shading ranging from green for lower expression in H- relative to L-gEBV fish, to red for higher expression in in H- relative to L-gEBV fish). All differences are significant. For genes that are represented by several paralogs, the number of paralogs are indicated in parentheses. All differentially expressed genes are shown in Supplementary Table 1.

One hundred and seventy-seven genes, including a panel of immune genes, were differentially expressed between H-gEBV and L-gEBV families in naïve fish. Higher expression in L-gEBV was found for several genes involved in early antiviral responses. These genes included *beta-2-microglobulin*, a component of MHC class I molecule, and four genes identified as virus responsive genes (VRGs^39^), including *ubiquitin protein ligase e3a* and two genes for *tripartite motive proteins (trim*, which most likely also interact with ubiquitin) (Fig. 5). *Mmp13* is an extracellular matrix degrading enzyme, which is consistently activated in pathogen infected Atlantic salmon. Immune genes with higher expression in H-gEBV Atlantic salmon heart included several segments of immunoglobulin and lectins. Differential expression (up to 12.8-fold difference between the groups) was observed in several genes encoding proteins with NACHT, LRR and PYD domains. The role of these salmon genes is uncertain, but mammalian proteins with similar architecture (NLRs) play a key part in innate immunity and inflammation^40,41^. A hallmark of naïve H-gEBV salmon was higher expression of a suite of genes with diverse roles in the neural system: patterning and differentiation (*pax-1, tomoregulin, endothelin-1 receptor*), neural transmission and synaptic functions (*discs large homolog, gaba a receptor, glutamate receptor associated, myelin protein*). Semaphorins regulate both neural and cardiac development^42^. Of note was higher level of gene expression for *c-type natriuretic peptide* in H-gEBV: these and related hormones are induced in the heart of virus infected Atlantic salmon^29,43^.

### Differential gene expression 4 weeks post-challenge

**Figure 6:**
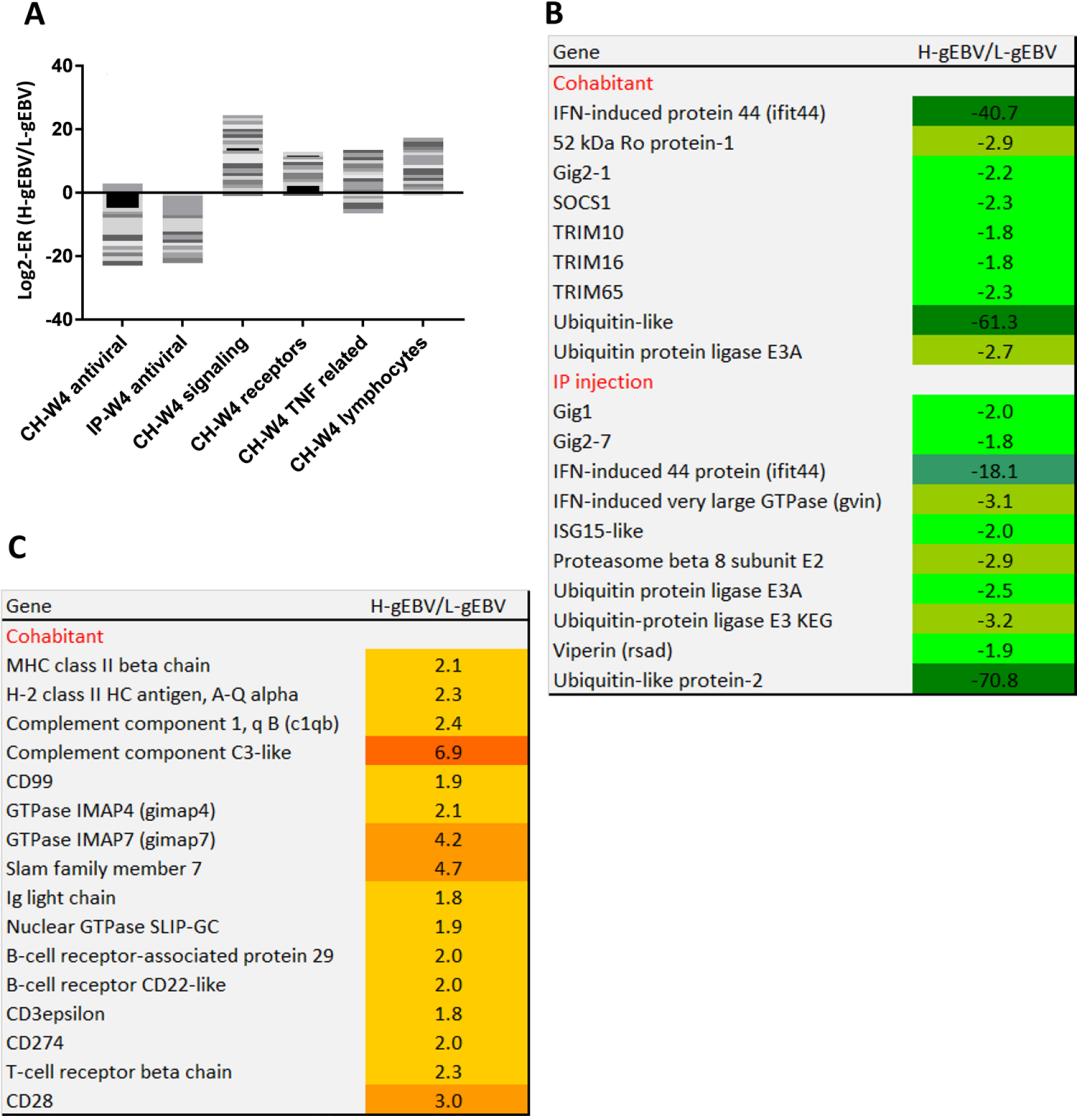
Highlighted immune genes of interest that were differentially expressed between the heart of fish with high and low genomic breeding values (H-gEBV/L-gEBV respectively) at W4 after PD challenge. (**A**) Stacked columns present H-gEBV to L-gEBV expression ratios (ER) for immune gene functional categories under the cohabitant (CH) and intraperitoneal injection (IP) challenge models, each band corresponds to a gene. (**B**) Virus responsive genes, VRG. (**C**) Genes involved in adaptive immune responses (CH challenge model).

Many immune genes showed differences between the H- and L-gEBV fish heart at W4 (Fig. 6A). In CH, a larger number of immune genes had higher expression in H- than L-gEBV fish heart (87 versus 30 genes), while the opposite was observed in IP (21 genes with higher and 47 genes with lower expression in H-gEBV). The time lag in the viral load and infection cycle with CH challenge may explain some of the differences in the gene expression patterns between the CH and IP trials. In both trials, a large proportion of the genes with lower counts of transcripts in H-gEBV (15 genes in both IP and CH trials) were known as virus responsive genes (VRGs) in Atlantic salmon^39^. These VRGs are known to be induced by many types of viruses. Several of the VRGs that were differentially expressed (Fig. 6B) are known to encode highly specialized proteins of innate antiviral immunity (e.g. *isg-15* and other ubiquitin related proteins*, rsad, gvin, ifit44*, members of *gig1* and *gig2* multigene families). Opposite profiles were observed for *matrix metalloproteinases 9* and *13*, which are potent inflammatory effectors that showed higher expression in H-gEBV in the CH trial and lower expression in the IP trial (Supplementary Table 1). Several functional groups of immune genes (chemokines, cytokines and their receptors, putative pathogen recognition receptors, TNF-related genes) had higher expression in H-gEBV than L-gEBV fish heart in CH (Fig. 6A). A tendency for for up-regulation of genes involved in acquired immune responses in H-gEBV than L-gEBV fish heart was observed in both trials, especially for CH (Fig. 6C). All viral infections induce genes involved in antigen presentation via MHCI (*components of mhcI* and *proteasome*), which are co-regulated with VRGs. Several genes of the MHCII complex were more highly expressed in H-gEBV than L-gEBV fish. Unlike the ubiquitously expressed MHCI, MHCII is specific for professional antigen presenting cells (for example, macrophages and B-cells). MHCII interacts with CD4 helper T-cells controlling differentiation of antigen-specific B-cells and cytotoxic T-cells. *Slamf 7* regulates differentiation of various lymphocyte lineages. *Gimaps* are essential for survival of naïve and activated B- and T-cells^44,45^. Several genes indicated higher activity of B-cells in H-gEBV. Nuclear *gtpase slip-gc* is expressed in mammalian germinal centres involved in the proliferation of B-cells^46^. *Cd22* or *siglec* are B-cell – specific adhesion molecules that regulate antigen receptor signalling^47^. Higher expression of *ig light chain* and *c1qb* was found in H-gEBV than L-gEBV fish. These are protein components that activate the classical complement pathway after binding to an antibody antigen complex. Greater stimulation of T-cells in H-gEBV than L-gEBV fish was indicated by differential expression of *T-cell receptors (tcr*) and several other emblematic marker genes. *Cd3* is a component of *tcr* and *cd28* provides costimulatory signals required for the survival and activation of T-cells. *Cd8* is specific for cytotoxic T-lymphocytes, which kill virus infected cells. In contrast, the difference between the salmon groups in the IP challenge model was much smaller, but several genes encoding components of *ig* showed higher expression in H-gEBV than L-gEBV fish, suggesting more active recruitment of B-cells (Supplementary Table 1).

**Figure 7:**
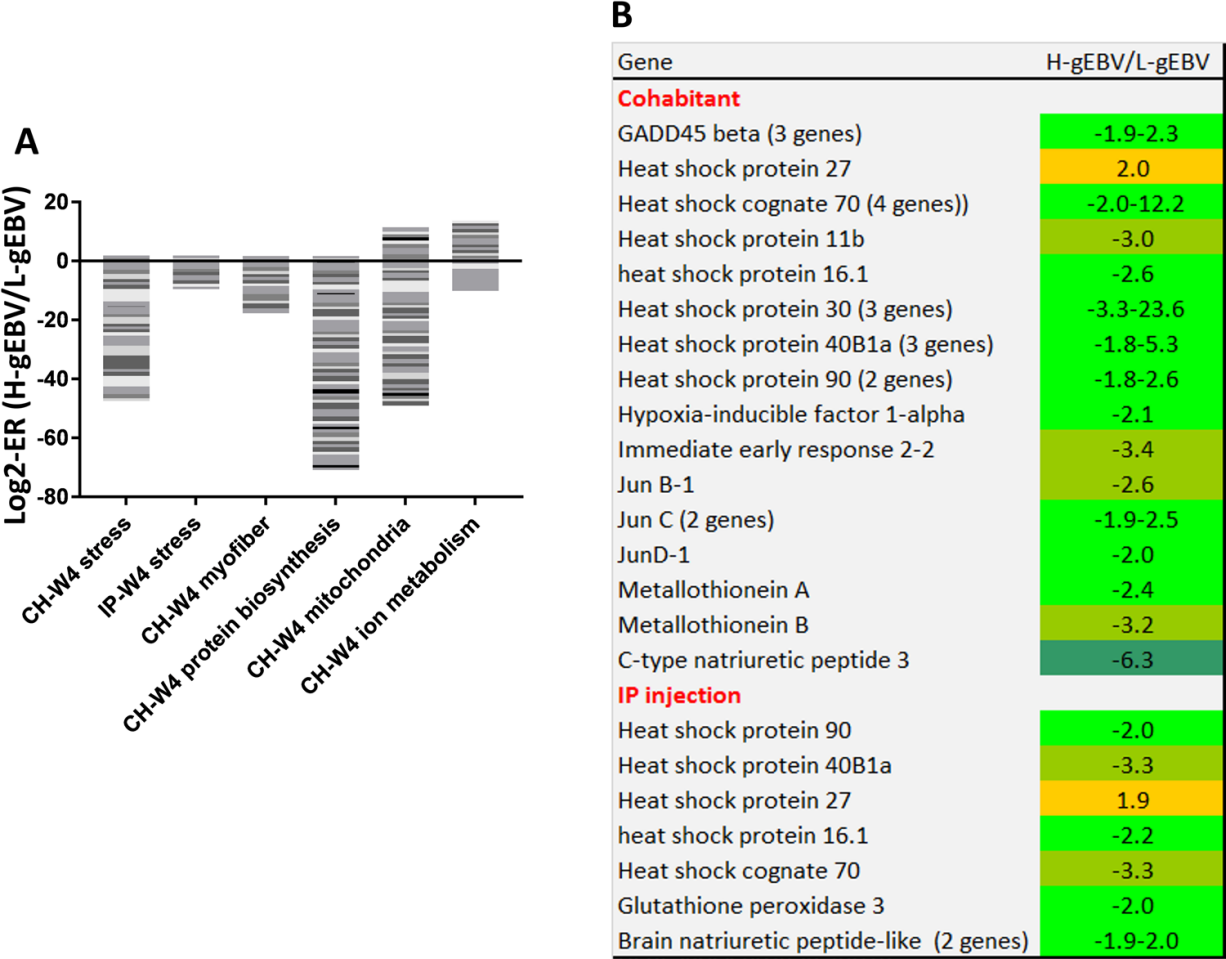
Highlighted stress response, metabolism and structural protein genes of interest with expression differences between fish with high and low genomic breeding values (H-gEBV and L-gEBV respectively) at W4 after PD challenge. (**A**) Stacked columns present H-gEBV to L-gEBV expression ratios (ER) under the cohabitant (CH) and intraperitoneal injection (IP) challenge models, each band corresponds to a gene. (**B**) Genes involved in stress responses.

H-gEBV fish were characterised by a weaker stress response to infection with SAV3 than in L-gEBV fish at W4 for both challenge models (Fig. 7A). Multiple genes could be categorised functionally as stress markers (Fig. 7B): chaperones (*heat shock proteins* and *cognates*), antioxidant enzyme *glutathione peroxidase* and metal binding proteins *metallothioneins*. Transcription factors *junb*, *c* and *d* and *immediate early response 2* consistently respond to a diverse range of stressors in Atlantic salmon. Fish with a high-gEBV were also associated with lower expression of myofiber proteins, multiple genes involved in protein biosynthesis (*ribosomal proteins, translation initiation* and *elongation factors*) and mitochondrial proteins with diverse functions (Supplementary Table 1). Three *natriuretic hormones* were down-regulated in H-gEBV with respect to L-gEBV: one gene in the CH and two genes in the IP trial (Fig. 7B).

### Differential expression 10 weeks post challenge

**Figure 8:**
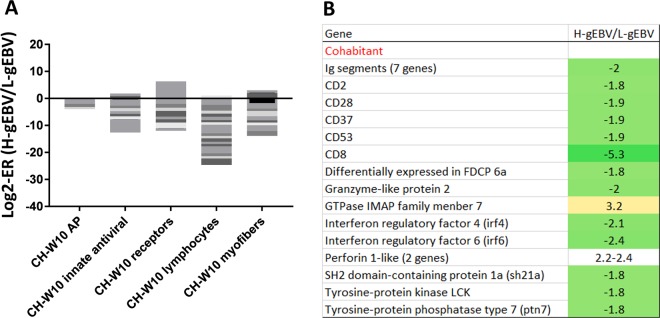
Highlighted genes of interest with expression differences between high and low genomic breeding value fish heart (H-gEBV and L-gEBV respectively) at W10 after cohabitant (CH) challenge. (**A**) Stacked columns present H-gEBV to L-gEBV expression ratios (ER) under the cohabitant (CH) and intraperitoneal injection (IP) challenge models, each band corresponds to a gene. (**B**) Genes involved in immune and stress responses.

In the CH trial, a larger number of immune genes showed lower expression in H-gEBV than L-gEBV fish heart at W10 (63 versus 16 genes) and this trend was observed for genes affecting both innate (mainly VRGs) and acquired immunity (Fig. 8A,B). Lower counts of transcripts encoding *ig segments* in H-gEBV than L-gEBV fish suggests reduced recruitment of B-cells to hearts infected by the virus in the H-EBV fish. Several genes regulate the differentiation and activity of lymphocytes. *Lck* controls the selection and maturation of T-cells, interactions of CD4 and CD8 with MHCII and MHC1 and signal transduction from T-cell receptors^48,49^. Multifunctional cell surface glycoprotein cd37 regulates the differentiation of T-cells^50^ and survival of plasma B-cells^51^. *Ptn7* plays role in T and B-lymphocyte development and signal transduction, *SH2 domain-containing protein 1 A* enhances NK cells cytotoxicity. *Cd2* is an adhesive and co-stimulatory molecule located on surface of T and NK cells and *cd226* is a costimulatory receptor^52^. With respect to cytotoxic effectors, two genes encoding *perforins*, pore forming molecules that open holes in the infected cell, showed higher expression in H-gEBV, however *granzyme* an extracellular serine protease released from T- and NK-cells was down-regulated in H-gEBV with respect to L-gEBV. In IP, only 22 immune genes were differentially expressed and numbers with higher and lower expression in H-gEBV were equal (Supplementary Table 1). Compared to L-gEBV, H-gEBV showed lower expression of a suite of genes with essential roles in cardiac functions: myofiber proteins in CH, *muscarinic cholinergic receptor 2B* and *C-type natriuretic peptide 3* in IP.

### Real-time qPCR

**Figure 9:**
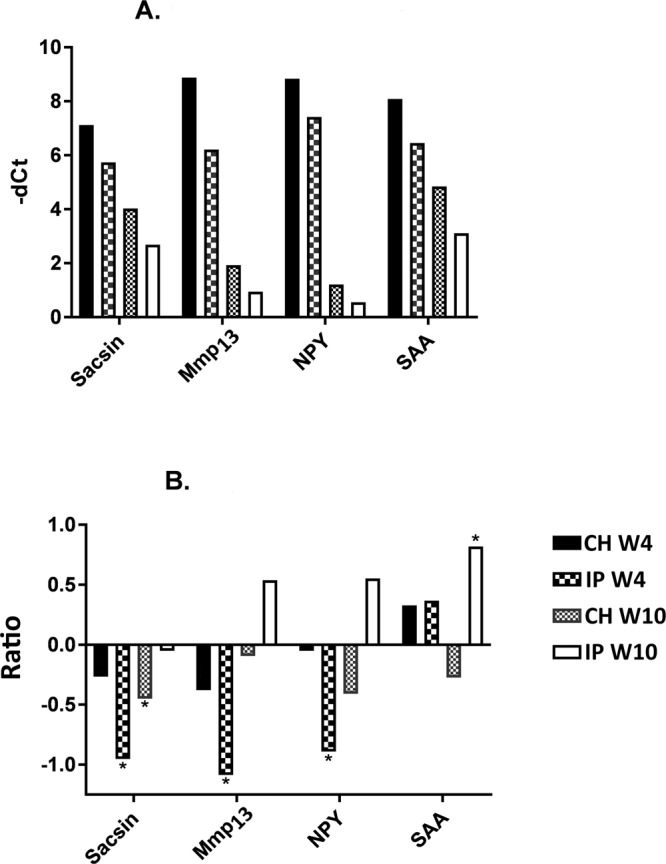
Relative gene expression (mean RT qPCR -dCt values) for the functional candidate genes chosen. (**A**) Challenge tested relative to naïve. Challenge with SAV3 up-regulated the expression of all analysed genes (within each gEBV group). Differences between the challenge models (CH and IP) and time points (W4 and W10) are significant with respect to naïve fish. (**B**) Comparison between high and low genomic breeding value fish (H-gEBV and L-gEBV respectively); all significant differences are indicated with * (p < 0.05).

One of the aims of our study was to test whether the pattern of gene expression for a small subset of immune genes known to respond to PD infection could be used to predict a salmon’s breeding value for PD resistance. If this were possible, such testing could substitute or reduce the need for pathogen challenge trials. Genes for RT-qPCR were selected based on data produced by several previous studies of PD^30,53,54^. All genes chosen for developing the diagnostic test panel for resistance were up-regulated after challenge with SAV3, confirming the results of our previous experiments, and the increase in expression was consistently greater in CH (Fig. 9A). At W10, expression decreased but remained higher than in uninfected controls, in concordance with clearance of the pathogen. Strong correlations were detected between viral load and gene expression for the functional candidate genes (*sacsin*, *matrix metalloproteinase 13*, *neuropeptide Y* and *serum amyloid A*, Pearson r^2^ of 0.85–0.91, p < 0.001). Each of the analysed genes could therefore be useful for the detection of viral infection in Atlantic salmon.

However, the genes that we targeted for RT-qPCR were unable to discriminate Atlantic salmon by genomic breeding value. No difference between L-gEBV and H-gEBV groups was found for naïve fish (data not shown). After challenge with the virus, each gene was significantly differentially expressed between the H-gEBV and L-gEBV groups for at least one time-point (Fig. 9B). However, discriminant analysis was unable to classify individuals as L-gEBV or H-gEBV based on the qPCR data. Results produced in this study suggest that RT qPCR analyses of small diagnostic gene sets might not be suitable for the discrimination of groups of fish with relatively small differences in resistance, and that diagnostic power is not enhanced with increased numbers of animals for analysis. One the main reasons is functional redundancy – disease resistance traits are typically polygenic and multiple genes have similar roles. Indications of functional redundancy were apparent in the current study. Functional groups showed similar changes in both trials: for instance, at W4, H-gEBV was characterized by a lower activity of genes with functions affecting innate antiviral immunity and higher expression of genes with functions affecting acquired immunity, but there was little overlap between the lists of differentially expressed genes for the CH and IP challenge models (Supplementary Table 1). The gene expression response of the fish depends partly on its genetic background (indicated by gEBV) and partly on the fishes’ history (e.g. prior exposure to the virus and other stresses during development etc.). The heritability of PD resistance in Atlantic salmon is moderate to high^23,24^, so life history is likely to have a substantial effect on resistance. Gene expression profiles have the potential to provide detailed phenotypes and could provide a means to discriminate between underlying genetic differences and differences in the stress history that contribute to an animal’s overall resistance to PD. However, the expression of genes in response to infection varies over time, and therefore a “snapshot” of gene expression will give different results depending on the timing and extent of infection and immune response at that moment in each fish. Gene expression profile data can be valuable when trying to identify causative genetic variants affecting resistance to the disease (e.g. by looking for allele specific expression or eQTLs that may be contributing to resistance^55^). The trends in differential expression observed in the RT qPCR results were confirmed by comparison to FPKM box plots generated from the mRNA-seq data for these same genes.

### Processes associated with resistance to PD and implications for aquaculture

The study has contributed to understanding the processes influencing protection against PD in Atlantic salmon. The results support our expectations that the immune response, or the functional performance of the cardiovascular system (e.g. heart: contraction and mechanical force, structure, developmental processes, metabolism and endocrine regulation), have major influences on the inherent ability of Atlantic salmon to resist disease. Several immune genes were differentially expressed between L-gEBV and H-gEBV fish heart before challenge with SAV3 (i.e. in naïve fish). Immune responses in Atlantic salmon usually presume co-ordinated expression changes of multiple genes with related roles; here in naïve fish, the functional groups and pathways of the immune system were represented with one or a few differentially expressed genes. Several genes showed higher expression in naïve and challenged H-gEBV salmon but only one gene, *alpha cardiac actin*, was noted in all comparisons. With respect to functional fitness, both GO enrichment analysis (Supplementary Table 2) and inspection of individual genes marked several functional categories associated with development of the cardio-neural system, indicating that neural regulation of the heart can be important for protection against the virus. After infection, transcriptomic analyses in challenged salmon suggested a strong immune response. Higher expression of innate antiviral genes in L-gEBV was in concordance with previous studies: expression levels of VRGs are correlated to pathogen loads (being consistently higher in the most infected individuals^25,39,56^). The resolution of viral diseases in Atlantic salmon normally takes place soon after the mounting of an adaptive immune response, as evidenced by the increased level of transcripts encoding B and T cell markers in infected hearts^56,57^. The gene expression profiles suggested earlier activation of both B- and T-cells in H-gEBV salmon, and this might be of crucial importance for the outcome of disease. Better protection against SAV3 might explain the lower expression of multiple stress genes in H-gEBV fish. At the same time, many genes with important cardiac functions showed higher expression in L-gEBV. The differential expression of genes encoding myofiber proteins was maintained between the H-gEBV and L-gEBV salmon groups, even at W10 when the disease was close to resolution. Cardiac hormone (*c-type natriuretic peptide*) showed higher expression in naïve H-gEBV than naïve L-gEBV salmon, but several natriuretic hormones were down-regulated with respect to L-gEBV after challenge. This might be due to the greater level of damage in the heart of the L-gEBV group fish.

The ability to discriminate Atlantic salmon with different breeding values from the relative expression of several marker genes might be difficult to achieve because the history of the fish influences how it responds to viral infection and clouds the underlying genetic differences between H-gEBV and L-gEBV fish. Whole transcriptome data could be potentially used^58^, but the cost-benefits of utilising whole transcriptome or multi-panel marker approaches needs to be thoroughly assessed^59^. Genes that were found to be differentially expressed between H-gEBV and L-gEBV fish before challenge from analysis of the transcriptome (eg. c-type lectin, NACHT, LRR or PYD domains, Fig. 5) could be potential markers, but expression differences would need to be confirmed in independent studies. Markers predicting the gEBV of naïve fish would be particularly useful as they would enable the use of non-destructive sampling and testing to predict the breeding value for PD resistance of candidates for selective breeding in the breeding nucleus. For aquaculture producers to also benefit, testing needs to be feasible to apply (and economically attractive) for screening a large number of individuals. It is also important to know whether the same responses that we observe under controlled challenge test conditions in tanks are observed with natural outbreaks of the disease in the field (cages in the sea), so further studies are needed to validate the applicability of these findings to the farm environment.

The gene expression results support the findings of other studies showing that the immune response, especially acquired immunity, plays a leading role in providing resistance to PD^29^. Further work is needed to confirm the associations with PD resistance and to determine whether these genes, or their cis- or trans-regulators of expression are associated with mapped QTLs for PD resistance.

### Summary

At week 4 post-infection, the heart of H-gEBV fish was less severely damaged by the intraperitoneal injection challenge than the heart of L-gEBV fish. Gene expression differences between H-gEBV and L-gEBV were enhanced in the co-habitant challenge model, and this model resulted in higher salmonid alphavirus subtype-3 loads than the intraperitoneal injection challenge model, but this could be due to differences in the timing of infection under the different challenge models. H-gEBV fish had a stronger and more diverse immune response involving signalling and communication (cytokines, chemokines and receptors), lectins, B- and T-cell genes. Further, they showed less signs of stress and had a lower antiviral response at week 4 than L-gEBV fish. At week 10 post-challenge, the H-gEBV fish immune system recovered better and faster (lower expression of innate, antiviral, B- and T-cell immune genes) than L-gEBV fish. The differential patterns of gene expression in H- and L-gEBV fish highlighted by this study provide a signature that could be useful for the prediction of breeding values for disease resistance, and the role of these genes and their effect on disease resistance warrants further investigation.

## Supplementary information

Dataset 1.
